# Comparing coagulation activity of *Selaginella tamariscina* before and after stir-frying process and determining the possible active constituents based on compositional variation

**DOI:** 10.1080/13880209.2017.1421673

**Published:** 2018-01-02

**Authors:** Qian Zhang, Ya-Li Wang, Die Gao, Liang Cai, Yi-Yao Yang, Yuan-Jia Hu, Feng-Qing Yang, Hua Chen, Zhi-Ning Xia

**Affiliations:** a School of Chemistry and Chemical Engineering, Chongqing University, Chongqing, PR China;; b School of Pharmacy, Southwest Medical University, Luzhou, PR China;; c State Key Laboratory of Quality Research in Chinese Medicine, Institute of Chinese Medical Sciences University of Macau, Macao, PR China

**Keywords:** Haemorheology and plasma coagulation system, natural products, activity transformation, dihydrocaffeic acid, amentoflavone

## Abstract

**Context**: *Selaginella tamariscina* (P. Beauv.) Spring (Selaginellaceae) (ST) has been widely used in China as a medicine for improving blood circulation. However, its processed product, *S. tamariscina* carbonisatus (STC), possesses opposite haemostatic activity.

**Objective**: To comprehensively evaluate the activity of ST and STC on physiological coagulation system of rats, and seek potential active substances accounting for the activity transformation of ST during processing.

**Materials and methods**: The 75% methanol extracts of the whole grass (fine powder) of ST and STC were prepared, respectively. Male Sprague–Dawley rats were randomly divided into five groups: control group, model group, model + ST group, model + STC group and positive control group (model + Yunnanbaiyao). The duration of intragastric administration was 72 h at 12 h intervals. Haemorheology parameters were measured using an LB-2 A cone-plate viscometer and the existed classic methods, respectively. SC40 semi-automatic coagulation analyzer was employed to determine coagulation indices. Meanwhile, HPLC and LC-MS were applied for chemical analyses of ST and STC extracts.

**Results:** STC shortened tail-bleeding time, increased whole blood viscosity (WBV) and plasma viscosity (PV), decreased erythrocyte sedimentation rate blood (ESR), reduced activated partial thromboplastin time (APTT) and increased the fibrinogen (FIB) content in the plasma of bleeding model rats. Although ST could shorten APTT and TT, the FIB content was significantly decreased by ST. Dihydrocaffeic acid with increased content in STC vs. ST showed haemostatic activity for promoting the platelet aggregation induced by collagen and trap-6, and reducing APTT and PT significantly with a concentration of 171.7 μM *in vitro*. Amentoflavone with reduced content in STC vs. ST inhibited ADP and AA-induced platelet aggregation significantly with a concentration of 40.7 μM.

**Discussion and conclusions:** As the processed product of ST, STC showed strong haemostatic activity on bleeding rat through regulating the parameters involved in haemorheology and plasma coagulation system. Two active compounds, dihydrocaffeic acid and amentoflavone, might be partially responsible for the haemostatic and anticoagulant activity of STC and ST, respectively.

## Introduction

Anticoagulation and procoagulation are two significant physiological processes. The normal coagulation pathway represents a dynamic balance between these two processes (Davie et al. 1991; Dahlback 2000). However, due to some genetic or acquired factors, disturbances of the natural balance between the procoagulation and anticoagulation processes would occur, which may finally result in a tendency of either serious bleeding or thrombosis (Mackman 2008). Imbalance of the coagulation system may occur in the perioperative period or during critical illness, which may be secondary to numerous factors leading to a tendency of either thrombosis or bleeding.

Many natural products derived from traditional Chinese medicines (TCMs) showed their therapeutic potentials in the treatment of bleeding or thrombosis (Chen et al. 2015; Xu et al. 2015; Xie et al. 2017; Wang et al. 2017). *Selaginella tamariscina* (P. Beauv.) Spring (ST) (Selaginellaceae), locally named ‘Juan Bai’, is widely distributed in China. ST was reported to have effective anticancer (Jung et al. 2017), antioxidative (Farombi and Owoeye 2011), antiinflammatory (Suh et al. 2010), antidiabetic (Nguyen et al. 2015a, 2015b), antiskin aging (Lee et al. 2008) and antibacterial (Lee et al. 2009) activities. Additionally, ST showed good anticoagulant effects to dissipate blood stasis, and thus clinically used to treat abnormality in haemorheology, such as cerebral ischaemia, myocardial ischaemia and diabetic complications in China (Yang and Flaws 1998). Different types of compounds, mainly including biflavonoid, lignans, selaginellins, phenolics, alkaloid and xylogen were found in ST (Weng and Noel 2013). The flavonoids (e.g., amentoflavone, hinokiflavone, sotetsuflavone and apogenin) were considered to be the major active constituents in *Selaginella* followed by phenylpropanoids (Zhang et al. 2011; Chen et al. 2016).

In China, carbonisatus products of TCMs, produced by heating to turn a burned black or burned brown surface but keep a brown or pale brown interior, were widely used in clinics for the treatment of bleeding diseases (Committee, NP 2015). *S. tamariscina* carbonisatus (STC), the carbonized product of ST through stir-frying showed obviously enhanced antihaemorrhagic activity (Peng et al. 2000; Yang et al. 2015). Although there were records about the characteristic of ST’s activity transformation associated with blood coagulation system, modern comprehensive pharmacological experiments were seldom conducted. Meanwhile, even though it was reported that the concentration of some flavones decreased after stir-frying (Li et al. 2011), no study revealed the specific compounds.

In the present study, the effects of ST and STC on some key parameters involved in haemorheological and coagulation system were compared through *in vivo* experiments. Furthermore, chemical variation of ST and STC was analyzed by HPLC and LC-MS. In addition, two potential active compounds dihydrocaffeic acid and amentoflavone were selected to verify their procoagulant and anticoagulant effects, respectively.

## Materials and methods

### Chemicals and reagents

Dihydrocaffeic acid and amentoflavone with 98% purity (determined with HPLC) was provided by PUSH Bio-technology Co., Ltd. (Chengdu, China). Platelet agonist thrombin (THR), arachidonic acid (AA) and collagen were obtained from Sigma (St. Louis, MO). Adenosine diphosphate (ADP) was the product of Wuhu Huaren Technology Co., Ltd. (Wuhu, China). Thrombin receptor activator peptide 6 (trap-6) was purchased from Nanjing Peptide Biotech Ltd. (Nanjing, China). Heparin sodium salt (185 USP units/mg) was bought from Shanghai Aladdin BioChem Technology Co., LTD (Shanghai, China). HPLC-grade acetonitrile and formic acid were obtained from Beijing InnoChem Science & Technology Co., Ltd. (Beijing, China). Yunnanbaiyao (YNBY) was a product of YunNanBaiYao Group Co., Ltd. (Kunming, China). Coagulation assay kit was purchased from Taizhou Steellex Biotechnology Co., LTD. (Taizhou, China). The water used for all the experiments was purified by water purification system (ATSelem 1820 A, Antesheng Environmental Protection Equipment Co., LTD., Chongqing, China). Unless otherwise specified, all other chemicals and solvents were guaranteed reagent grade and purchased from Sigma (St. Louis, MO).

### Plant material

The whole grass of *S. tamariscina* was purchased in December 2016 from Beijing Tongrentang Medicine Corporation Ltd. (Chongqing, China), and was authenticated by Professor Fengqing Yang (School of Chemistry and Chemical Engineering, Chongqing University). Voucher specimens (No. ST2016112001) were deposited at the Pharmaceutical Engineering Laboratory in School of Chemistry and Chemical Engineering, Chongqing University, Chongqing, China.

### Sample preparation and extraction

The ST materials were oven-dried at 35 °C for 48 h, and then cut into small pieces. After that, it was weighed a certain amount of raw medicine for STC preparation. STC was prepared by stir-frying on the basis of the relevant standards for carbonizing (article 0213 general rule for medicinal process) in Chinese Pharmacopoeia (2015 edition) (Committee, NP 2015).

Afterward, all the dried raw ST and STC were pulverized and griddled through 50 mesh sieves (about 0.29 mm) prior to extraction. The fine powder (100 g) was transferred to a glass-stoppered conical flask (1 L) separately, and then 800 mL 75% methanol was added and sonicated for 20 min under room temperature. The mixture was reflux extracted in 80 °C water bath for 1 h, and the supernatant was obtained by filtration. The residue was collected and extracted repeatedly by the above process for three times. The combined filtrates were evaporated in a rotavapor (ZFQ 85 A, Shanghai Medical Instrument Special Factory, Shanghai, China) at 45 °C under reducing pressure to remove solvents. Finally, the extracts were further dried at 45 °C under vacuum (DZF-6050, Shanghai Jing Hong Laboratory Instrument Co., Ltd., Shanghai, China) to obtain the 75% methanol extract of ST and STC with yields of 21.6% and 19.2%, respectively. For *in vivo* test, the dried extract was dissolved in 0.5% CMC-Na solution with a concentration of 100 mg/mL. The extracts were dissolved in methanol (10 mg/mL for both ST and STC), and filtered through a 0.22 μm nylon membrane (Shanghai Titan Scientific Co., Ltd., Shanghai, China) for further HPLC and LC-MS analyses.

### Animal treatment

Male Sprague–Dawley (SD) rats (200–220 g) and rabbits (2–2.5 kg) were provided by the Animal Centre of Chongqing Medical University (Chongqing, China). They were housed in cages with unrestricted access to food and water with a constant temperature (ca. 25 ± 1 °C) and humidity (ca. 60 ± 2%) and with a 12 h light/dark cycle. All animals underwent an adaptation period of 7 days. Before the experiments, no food was given for 12 h, while water was available all the time. All experimental procedures were approved by the Institutional Animal Ethical Committee of Chongqing University, and were conducted according to the Guide for the Care and Use of Laboratory Animal of the National Institute of Health (Publication No. 80-23, revised 1996).

### Experimental group design

Thirty rats were randomly divided into five groups (six in each). Group 1 was the control one: rats were given the same volume of blank solvent (0.5% CMC-Na) as the vehicle. Group 2 was the model one: rats were given the same volume of blank solvent. Group 3 was model + ST one: rats were given 1.0 g/kg ST extract. Group 4 was model + STC one: rats were given 1.0 g/kg STC. The dosage of ST and STC was translated and calculated from the conversion coefficient table for animal and human weight [0.2 g ST crude herb/kg ×6.25 × 4 × 20% yield (w/w, dried extract/crude herb)]. Group 5 was model + YNBY one: rats were given 300 mg/kg YNBY. All treatments were performed by intragastric administration and were administered seven times with an interval of 12 h.

The rats in the control group were injected with 0.9% (w/v) NaCl saline solution, and all other rats were injected subcutaneously with heparin (500 U/kg) 5 min after the last administration. Rats were anaesthetized with 1.5% pentobarbital sodium (200 mg/kg) 50 min after the injection of heparin, and blood samples were collected after testing bleeding time.

### Bleeding time assay

After the injection of pentobarbital sodium, the bleeding time was measured immediately. The tail-bleeding model was conducted on the basis of the previous method with slight modifications (Stenberg et al. 1998). Briefly, the tails of rats were transfected with a sterile razor blade at the site that 5 mm apart from the tip. The resultant wound was gently blotted by filter paper with every 30 s. The bleeding time was the time from the start of transection to bleeding cessation. The time without outflow of blood for 30 s was considered as bleeding cessation. Pressure was applied to the wound to stop bleeding, if the bleeding time was greater than 30 min.

### Blood collection

After anaesthetized with pentobarbital sodium, blood was drawn from the abdominal aorta of rats to determine haemorheological variables. Blood was collected and anticoagulated with 3.2% sodium citrate (citrate/blood: 1/9, v/v), used for detection of whole blood viscosity (WBV) and packed cell volume (PCV). Additional 3.2% sodium citrate was added to the whole blood to make a 25% citrate solution (citrate/blood: 1/4, v/v), and was used for erythrocyte sedimentation rate blood (ESR). Then, plasma was separated by centrifugation at 2259 *g* for 10 min for detection of plasma viscosity (PV) and plasma anticoagulation. All experiments were completed within 3 h after blood collection.

### Viscosity determination

Blood or plasma (800 μL) was used to determine the viscosity under 37 °C with a cone-plate viscometer (Model LB-2 A, Tianjin Tangyu Medical Technology Development Co., Ltd., China) at different shear rates. WBVs were measured with shear rates, 10, 80 and 150 S^−1^; PV was measured at 100 S^−1^ shear rate.

### ESR and PCV measurement

Blood (1 mL with 25% of 3.2% sodium citrate) was put into Westergren’s blood sedimentation tube to observe the rate of red blood cells falling to the bottom of the tube (mm/h). Appropriate amount of anticoagulated whole blood was collected in the 10-scale tube and aligned the concave surface with the ‘0’ scale. Then, the tube was centrifuged at 2259 *g* for 30 min. After that, the tube was taken out and placed on table under room temperature. Finally, the PCV was read after 5 min.

### Plasma anticoagulation assay

Thrombin time (TT), prothrombin time (PT), activated partial thromboplastin time (APTT) and fibrinogen content (FIB) were examined on the basis of the manufacturer’s instructions with commercial kits by a coagulometer (SC40 semi-automatic coagulation analyzer, Taizhou Steellex Biotechnology Co., LTD., China). TT was determined by incubating 100 μL plasma solution for 3 min at 37 °C, followed by addition of 100 μL thrombin agent. PT was determined by incubating 50 μL plasma solution for 3 min at 37 °C, followed by addition of 100 μL thromboplastin agent. PT results were expressed as International Normalized Ratios (INR) [INR = (PT sample/PT control) ISI, ISI = international sensitivity index]. APTT was determined by incubating 50 μL plasma with 50 μL APTT-activating agent for 3 min at 37 °C, followed by addition of 50 μL CaCl_2_. FIB was determined by incubating 10 μL plasma with 90 μL imidazole buffer for 3 min at 37 °C, followed by addition of 50 μL FIB agent.

### HPLC and LC-MS analyses

HPLC analysis was performed on an Agilent 1260 Series liquid chromatograph system (Agilent Technologies, Palo Alto, CA), which was equipped with a vacuum degasser, a binary pump, an auto-sampler and a diode array detector (DAD), controlled by an Agilent ChemStation software. An Agilent ZORBAX SB-C_18_ column (250 × 4.6 mm i.d., 5 µm) along with a pre-column (ZORBAX SB-C_18_ guard column, 12.5 × 4.6 mm i.d., 5 μm) were employed. The separation was achieved by a gradient solvent system consisted of solvent A (0.1% formic acid solution) and solvent B (acetonitrile) as mobile phases. And, the gradient elution profile was: 0–5 min, 10% B; 5–10 min, 10%–20% B; 10–40 min, 20%–40% B; 40–50 min, 40%–45% B; 50–60 min, 45%–55% B; 60–70 min, 55%–80% B; 70–75 min, 80%–90% B; 75–80 min, 90%–10% B. The solvent flow rate was 0.8 mL/min, DAD detection wavelength was set at 265 nm, the column temperature was kept at 30 °C, and the injection volume for all samples was 10 μL.

The LC-MS analysis was conducted on a Shimadzu LC-MS system (Shimadzu, Kyoto, Japan) consisting of a hybrid ion trap time-of-flight mass spectrometer (Shimadzu) equipped with an electrospray ionization (ESI) interface was connected to the LC system via a PEEK tube (0.13 mm i.d.) to perform high-resolution tandem mass spectrometry. Chromatographic separation conditions were the same as that of HPLC-DAD analysis. For the mass spectrometer, an accurate ion axis was calibrated using the sodium trifluoroacetate clusters as a reference. Nitrogen was used as the nebulizing and drying gas, whereas ultrahigh purity argon (Ar) was used as the cooling and collision gas for collision-induced dissociation (CID). The mass spectrometric parameters were set as below: ionization polarity, positive and negative; drying gas pressure, 100 MPa; curved desolvation line (CDL) voltage, constant level; interface voltage, 1.4 kV; nebulizing gas flow rate, 1.5 L/min; detector voltage, 1.40 kV; CDL temperature, 200 °C; block heater temperature, 200 °C; and IT vacuum, 1.9 × 10^−2 ^Pa. Positive mass spectra were recorded in the full scan and automatic multiple stage fragmentation scan modes over a range of *m/z* 100–1000 for all MS^1^ and MS^2^ spectra acquisition. The ion accumulation time was set at 100 ms and the collision energy of CID was set at 50%. Data acquisition and processing were performed with the LC-MS solution version 1.1 software package (Shimadzu). The accuracy of the assigned chemical formula was determined using a mass difference tolerance of ±5 ppm, which was calculated by the deviation between the experimental mass and calculated mass.

The peaks with significant differences in HPLC chromatograms of ST and STC were calculated according to the difference in peak area, and the relative percentage of variation was calculated according to the following formula:

$$\text{Δ}P\;\text{\%=}\frac{P_{\text{STC}}\text{-}P_{\text{ST}}}{P_{\text{ST}}}\times \text{100\%}\text{, }$$

where *P*_ST_ and *P*_STC_ are the specific peak areas in the chromatogram of ST and STC extract, respectively.

### Haemostatic activity tests of the predicted compounds

#### Platelet aggregation test in vitro

Rabbit blood samples were collected in 3.8% sodium citrate with the ratio of 9:1 (blood: anticoagulant) from carotid artery after anaesthetizing by 1% pentobarbital sodium. Platelet-rich plasma (PRP) was obtained by centrifugation at 93 *g* for 15 min at room temperature, and platelet-poor plasma (PPP) was obtained by further centrifugation from the remaining blood at 2325 *g* for 15 min. The concentration of PRP was adjusted to 3 × 10^11^/L by PPP.

The antiplatelet aggregation study was performed by turbidimetric method as described in previous report (Chen et al. 2017). Platelet aggregation agonist ADP (final concentration 10 μM) and trap-6 (15.625 μM) was prepared with 0.9% saline solution, collagen (0.1 μg/mL) and THR (0.25 U/mL) was dissolved in deionized water, AA (0.205 mM) was prepared with 1% NaHCO_3_ solution. PRP (300 μL) was pre-incubated with test sample solution (10 μL) or reference blank solution (10 μL) at 37 °C for 3 min before being stimulated with agonist (10 μL). Platelet aggregation was recorded for 5 min with stirring. Physiological saline was used as blank control. All tests were performed within 3 h after the collection of blood. Aggregations were measured and expressed as per cent changes in light transmission with respect to PPP. The percentage of aggregation inhibition was estimated by the formula: inhibitory (%) = (maximal aggregation of the blank control − maximal aggregation of compound-treated PRP) × 100%/maximal aggregation of the blank control, and the % of aggregation promotion is calculated oppositely. Data were expressed as means ± SD.

### Plasma pro-coagulation assay in vitro

The obtained PPP was used for the *in vitro* plasma procoagulation assay, and the test was carried out according to a reported method with minor modifications (Tang et al. 2017). PPP (50 μL) was mixed with 50 μL of a solution of purified reference compounds and incubated for 3 min at 37 °C, and subsequently, 50 μL pre-warmed APTT reagent was added, mixed and incubated for 3 min at 37 °C. After that, pre-warmed CaCl_2_ (50 μL) was added, and the APTT was recorded in blood coagulation analyzer. 80 μL of PPP was mixed with 20 μL of the reference compound solution and incubated at 37 °C for 3 min. 100 μL TT assay reagent was added and clotting time was recorded. For the PT assay, PPP (30 μL) was mixed with 20 μL reference compound solution and incubated for 3 min at 37 °C. Finally, 100 μL of pre-warmed PT reagent was added, and the time for clot formation was determined. During the PT assay, 0.9% NaCl solution was used as control.

### Statistical analysis

Data were expressed as mean ± SD, and analyzed by SPSS software (version 23, SPSS, Inc., Chicago, IL). Student’s *t*-test was performed to assess the statistical significance. *p* Value of less than 0.05 was regarded as statistically significant.

## Results

### Effects of ST and STC on tail-bleeding time

**Figure 1.**
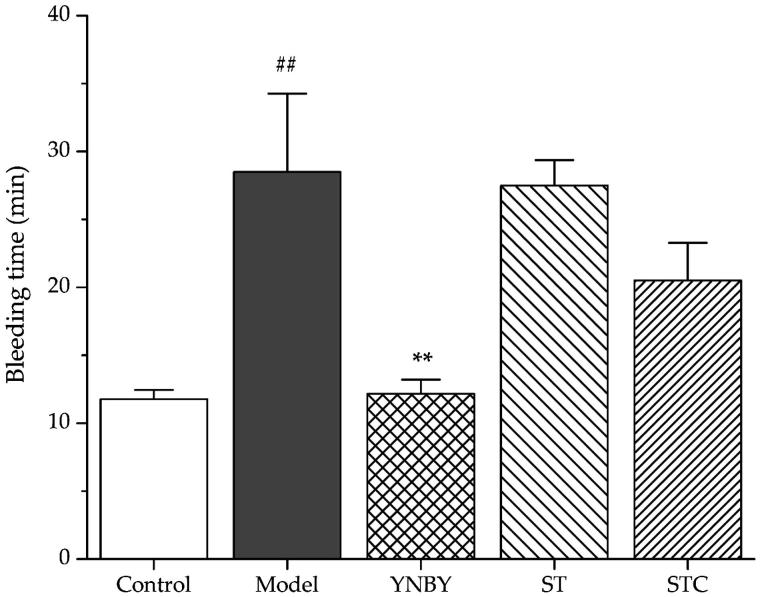
Effects of ST and STC on bleeding time. Data are shown as mean ± SD from six independent experiments (*n* = 6). #*p* < 0.05, ##*p* < 0.01 vs. control group. *p < 0.05, **p < 0.01 vs. model group.

The haemostatic effects of ST and STC were conducted by tail-bleeding time assay on rats. Control and heparin model rats were consecutively administered with corresponding extracts for 3 days as described in Experimental group design section. As shown in Figure 1, heparin (500 U/kg) significantly prolonged the bleeding time compared with control group (*p* < 0.01). YNBY (300 mg/kg) served as a positive control shortened bleeding time significantly (*p* < 0.01). ST and STC did not show obvious influence on rat’s bleeding time (*p* > 0.05).

### Effects of ST and STC on WBV and PV

To determine the effects of ST and STC on plasma viscosity, WBV and PV were measured. As illustrated in Table 1, WBV significantly (*p* < 0.01) decreased at all shear rates in model group and significantly increased in YNBY (positive group) and STC group after administration. However, WBV did not show apparent change in ST group with different shear rates (*p* > 0.05). Furthermore, the model group had significantly lower PV as compared with the control group (*p* < 0.05), and PV in YNBY and STC group significantly decreased as compared with the model group (*p* < 0.05).

**Table 1.** Effects of ST and STC on WBV and plasma viscosity at various shear rates (*n* = 6).

	WBV (mPa* s)			PV (mPa* s)
Group	150 s^−1^	80 s^−1^	10 s^−1^	100 s^−1^
Control	4.79 ± 0.04	5.80 ± 0.04	9.77 ± 0.08	0.63 ± 0.08
Model	4.04 ± 0.10##	4.82 ± 0.08##	8.39 ± 0.41##	0.43 ± 0.03#
YNBY	4.78 ± 0.15**	5.80 ± 0.20**	9.68 ± 0.20**	0.58 ± 0.08*
ST	4.19 ± 0.25	5.04 ± 0.25	8.56 ± 0.53	0.45 ± 0.07
STC	4.81 ± 0.16**	5.88 ± 0.24**	10.09 ± 0.62**	0.52 ± 0.03*

Data represent mean ± SD; *n* = 6.

# *p* < 0.05.

## *p* < 0.01 vs. control group.

* *p* < 0.05.

** *p* < 0.01 vs. model group.

### Effects of ST and STC on ESR and PCV

**Figure 2.**
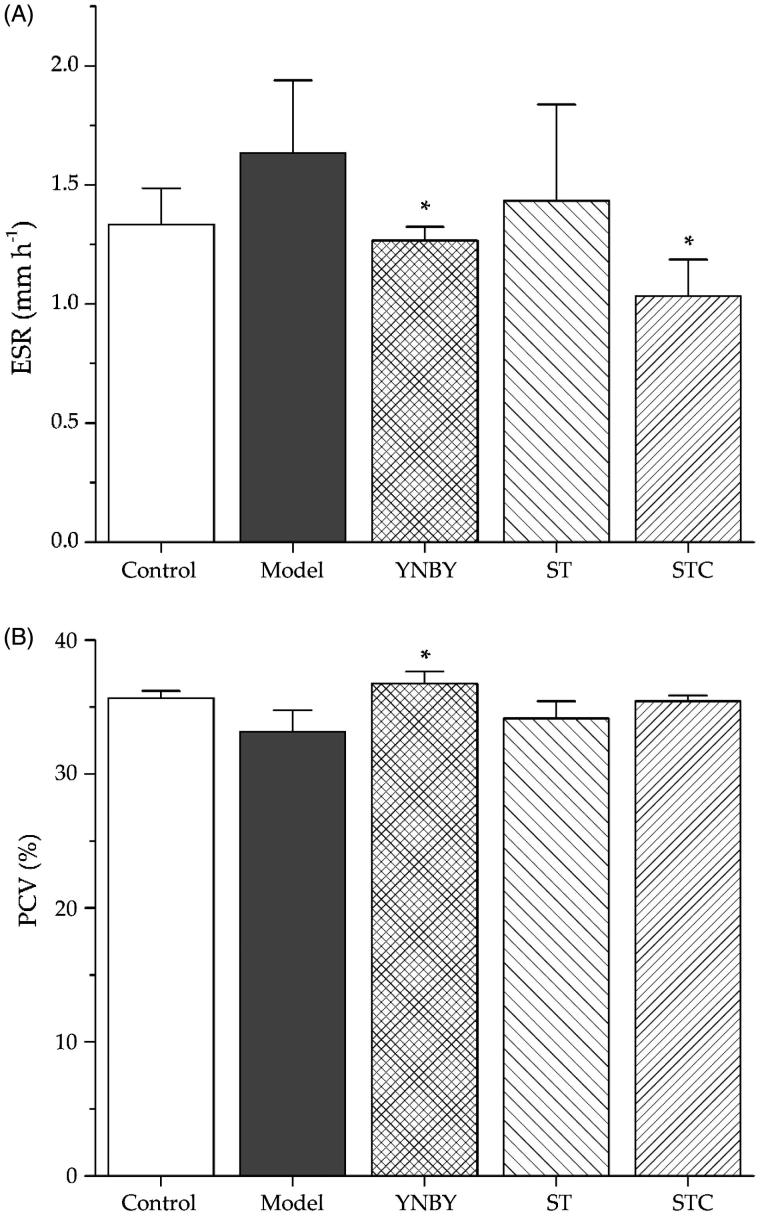
Effects of ST and STC on (A) erythrocyte sedimentation rate blood an d (B) packed cell volume . Data are shown as mean ± SD from six independent experiments (*n* = 6). #*p* < 0.05, ##*p* < 0.01 vs. control group. *p < 0.05, **p < 0.01 vs. model group.

The effects of ST and STC on ESR and PCV were investigated. As shown in Figure 2(A), ESR in the model group was higher than that in the control group, and YNBY and STC can lower the ESR in model group significantly (*p* < 0.05). As indicated in Figure 2(B), PCV in the model group was not obviously affected by the injection of heparin, as well as the administration of ST and STC, but YNBY can significantly increase PCV percentage (*p* < 0.05).

### Effects of ST and STC on plasma coagulation parameters

Plasma coagulation parameters were evaluated by measuring PT, APTT, TT and FIB *in vivo*. As shown in Table 2, in the model group, APTT and TT were prolonged, and PT (INR) increased significantly (*p* < 0.01). Comparing to YNBY group, which effected on all of the coagulation parameters significantly (APTT and TT were shortened, PT decreased with *p* < 0.01 and FIB content increased with *p* < 0.05), ST and STC groups had no obvious effect on PT (INR), but ST can significantly shorten APTT and TT (*p* < 0.05), while STC only affected on APTT (*p* < 0.05). In addition, STC elevated plasma FIB concentration significantly (*p* < 0.01), while ST could lower the FIB concentration (*p* < 0.01).

**Table 2.** Effects of ST and STC on plasma coagulation parameters (*n* = 6).

Group	PT (INR)	APTT (S)	TT (S)	FIB (g/L)
Control	1.32 ± 0.02	12.23 ± 0.21	21.70 ± 0.26	3.16 ± 0.39
Model	3.00 ± 0.18##	26.10 ± 2.05##	26.77 ± 0.23##	2.74 ± 0.12#
YNBY	1.10 ± 0.10**	10.47 ± 0.15**	20.70 ± 0.79**	4.24 ± 0.24**
ST	2.68 ± 0.03	21.78 ± 0.73*	24.57 ± 0.91*	1.93 ± 0.10**
STC	2.70 ± 0.16	20.70 ± 0.87*	25.17 ± 2.83	4.61 ± 0.10**

Data represent mean ± SD; *n* = 6.

# *p* < 0.05.

## *p* < 0.01 vs. control group.

* *p* < 0.05.

** *p* < 0.01 vs. model group.

### Analyses of ST and STC by HPLC and LC-MS

#### Seeking for major distinguishing compounds in the extract of ST and STC

**Figure 3.**
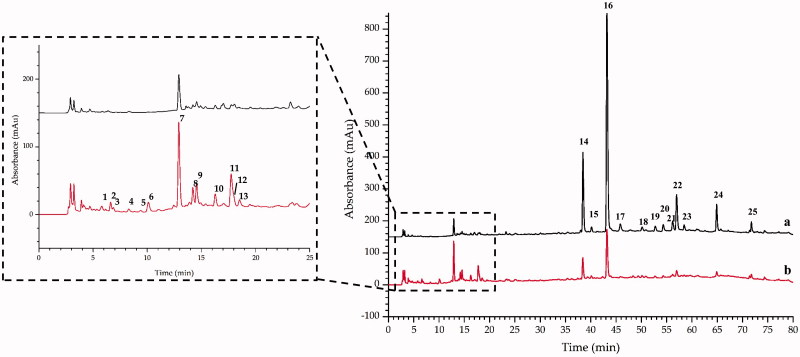
The representative HPLC-DAD chromatograms of (a) ST and (b) STC.

After optimized some experimental parameters such as the mobile phase system (methanol–water and acetonitrile–water), gradient elution program, type and amount of acid added to the aqueous phase, as well as detection wavelength, the chromatograms of ST and STC analyzed by HPLC-DAD are shown in Figure 3. The peak areas of the major constituents in ST and STC samples varied significantly. Under the same concentration, 25 peaks were indicated to be undergone changes during the stir-frying process. The average peak area (*n* = 3) and the percentage of change in peak area after processing is presented in Table 3. Peaks 2, 3, 5 and 6 were new peaks (compounds) appeared in STC (their peak areas were approximately equal to 0 in ST), and the peak areas of 1, 4, 7–13 were increased in STC compared with ST, but some peaks (peak 14–25) decreased after processing.

**Table 3.** Peak area of each common peak and their difference according to the percentage of the peak area (*n* = 3).

		Average peak area (mAu*s)^a^		
Peak No.	RT (min)	ST	STC	Relative peak area (%)
1	5.80	13.708	84.068	513.3
2	6.62	0	130.509	-^c^
3	6.86	0	54.734	-^c^
4	8.30	23.411	62.233	165.8
5	9.38	0	35.127	-^c^
6	10.10	0	216.239	-^c^
7	12.92	589.492	1437.932	143.9
8	14.21	49.023	291.345	494.3
9	14.55	134.170	385.475	187.3
10	16.28	57.813	218.027	277.1
11	17.75	76.123	-^b^	-^b^
12	18.06	82.090	-^b^	-^b^
13	18.50	24.553	173.790	607.8
14	38.44	4060.629	1111.537	−72.6
15	40.15	290.058	162.276	−44.1
16	43.20	13,261.2	2829.810	−78.7
17	45.84	695.155	125.190	−455.3
18	50.15	283.666	120.318	−135.8
19	52.75	360.707	130.619	−176.2
20	54.33	445.917	111.570	−299.7
21	56.19	617.787	196.478	−214.4
22	56.97	2281.816	379.666	−83.4
23	58.47	321.651	84.189	−282.1
24	64.89	1481.749	470.781	−68.2
25	71.75	495.447	209.253	−136.8

a Data are shown as mean from three independent experiments, *n* = 3.

b Value cannot be calculated accurately.

c The value is approximate infinity.

#### LC-IT-TOF-MS identification of the potential active compounds in STC

**Figure 4.**
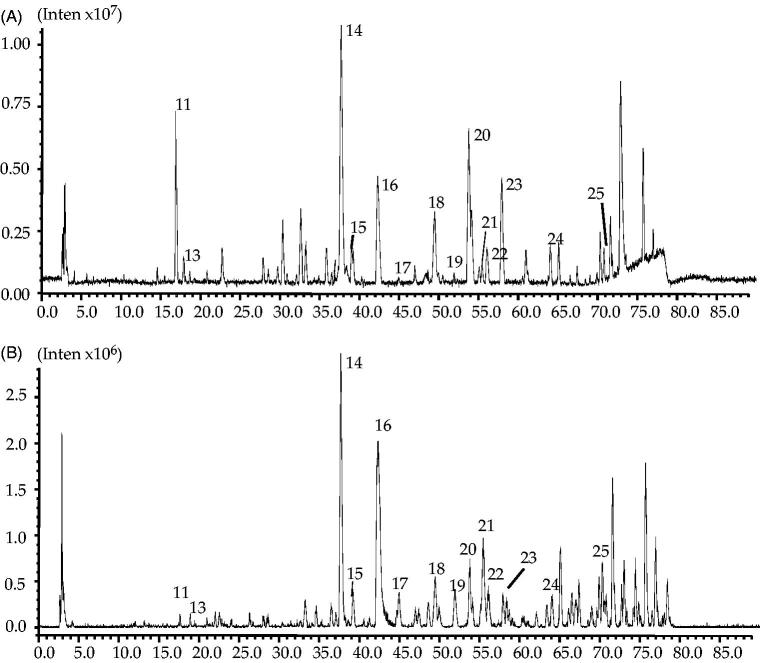
Total ion chromatograms of STC in (A) positive mode and (B) negative mode.

Since all 25 varied compounds (peaks) contained in the extract of STC, LC-IT-TOF-MS was employed to identify the potential active compounds in STC. Both negative and positive ion modes were tried, and the total ion chromatogram (TIC) is shown in Figure 4. It is clearly noted from Figure 3, most of the newly appeared peaks were the trace constituents in STC extract, and the low UV and MS responses caused certain difficulties for identification. After all, by comparing the retention time (tR), on-line maximum UV absorption wavelength (UV *λ*max (nm)) and MS data of the peaks with previous reports (Zhang et al. 2011; Weng and Noel 2013), 11 of 25 peaks were tentatively identified (Table 4). Two of the identified compounds include dihydrocaffeic acid (13) and amentoflavone (16) were selected, and their pure compounds were used for further pharmacological verification tests.

**Table 4.** Identification of constituents in ST and STC by HPLC–IT-TOF–MS in positive and negative ion mode.

Peak No.	tR	UV *λ*max (nm)	M + H^+^	MS^2^	M − H^−^	MS^2^	Molecular formula	Plausible identity
11	16.87		264.1011	236.1047; 170.0607				Unknown
13	17.89	220, 280	183.0639	155.0719; 123.0416	181.0576		C_9_H_10_O_4_	Dihydrocaffeic acid
14	37.65	265, 295	513.1727	467.1767; 419.1286; 378.1252	511.157	404.1059; 375.0987	C_34_H_24_O_5_	Selaginellin J
15	39.22	265, 300	513.1818	419.1394; 285.0859	511.156	481.1415; 404.1079; 389.1165; 375.1059; 297.0898; 197.0651	C_34_H_24_O_5_	Selaginellin I or iso
16	42.31	270, 335	539.1008	497.0921; 403.0459; 377.0677; 335.056	537.0825	443.041; 375.0514; 331.0612	C_30_H_18_O_10_	Amentoflavone
17	45.81	270, 340			537.0847	443.0397; 375.0515; 309.0396; 251.0327	C_30_H_18_O_10_	Hinokiflavone
18	49.45		511.1557	417.1129; 323.0679	509.1425	467.1301; 416.105; 374.0966		Unknown
19	51.92	265, 340	553.1165	521.0859; 401.1019; 376.0581; 255.0584	551.0993	519.0731; 457.0557; 431.085	C_31_H_20_O_10_	4′-O-Methyl robustaflavone
20	53.86	295, 430	483.1603	484.1625; 389.117; 371.1135; 271.0742	481.146	388.1095; 359.1099; 295.0751; 184.0481	C_33_H_22_O_4_	Selaginellin A
21	55.44	270, 340	553.116	511.11; 417.0598; 361.0703; 270.0549	551.1009	457.0565; 389.0664; 375.051	C_31_H_20_O_10_	7-O-Methyl robustaflavone
22	56.20	270, 340	539.0972	493.0933; 417.0628; 375.0536; 269.0474	537.0856	387.0858; 375.0557; 270.0507; 241.0449	C_30_H_18_O_10_	Robustaflavone
23	57.99	300, 430	497.1771	403.1332; 385.1223; 309.0901; 199.0748	495.1629	401.1186; 373.1207; 161.0425	C_34_H_24_O_4_	Selaginellin B or Selaginellin N
24	64.09	270, 340	553.12096	536.0734; 492.0879; 255.0304	551.0981	554.1175; 521.0884; 401.1039; 375.0468; 299.0553; 255.064; 135.0013	C_31_H_20_O_10_	7-O-Methyl amentoflavone
25	70.66	270, 330	581.1497	549.1209; 449.081; 431.0764; 298.0776	579.1302	547.1020; 403.0823	C_33_H_24_O_10_	Heveaflavone

### Haemostatic activity assay of dihydrocaffeic acid and amentoflavone

**Figure 5.**
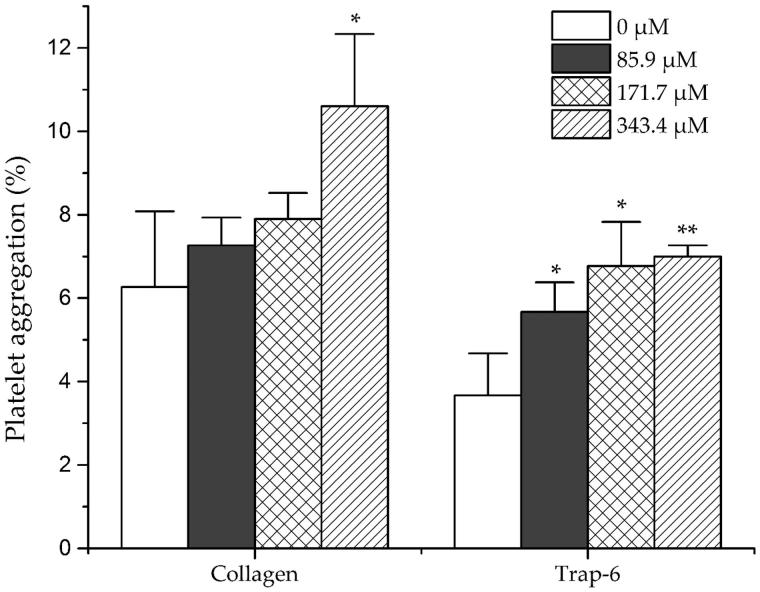
Effects of dihydrocaffeic acid on New Zealand rabbit platelet aggregation induced by collagen and trap-6. Data are expressed as mean ± SD of six measurements (*n* = 6). **p < 0.01 as compared to control group; *p < 0.05 as compared to control group.

**Figure 6.**
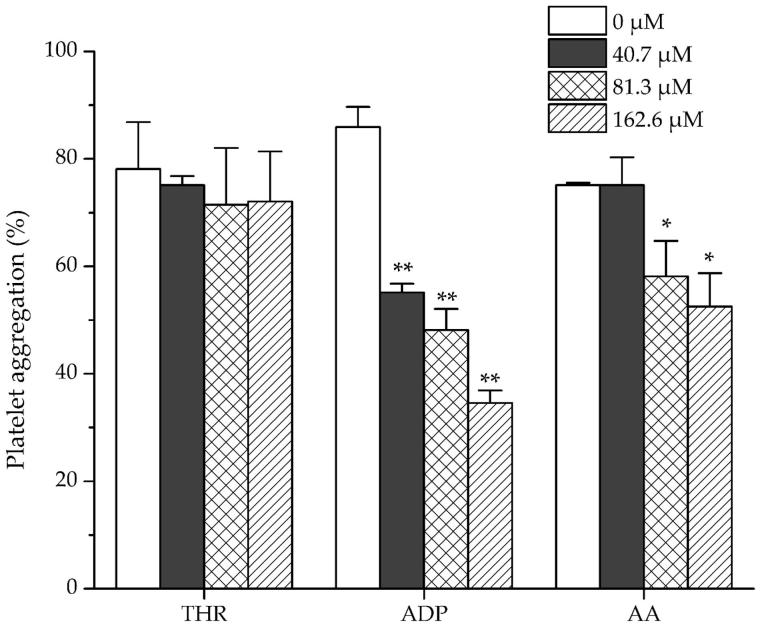
Effects of amentoflavone on New Zealand rabbit platelet aggregation induced by THR, ADP and AA. Data are expressed as mean ± SD of six measurements (*n* = 6). **p < 0.01 as compared to control group; *p < 0.05 as compared to control group.

The haemostatic activities of dihydrocaffeic acid were investigated by measuring the platelet aggregation induced by collagen and trap-6, and the influence on the coagulation parameters (TT, APTT and PT). As shown in Figure 5, dihydrocaffeic acid significantly promoted the collagen-induced platelet aggregation at high concentration (343.4 μM) and promoted the trap-6 induced platelet aggregation under relatively low concentration (85.9 μM). For coagulation parameters, as shown in Table 5 dihydrocaffeic acid could dose dependently decrease APTT and PT, but had no significant effect on TT. On the other hand, it was found that amentoflavone showed no effect on THR-induced platelet aggregation, but it could inhibit the platelet aggregation induced by ADP strongly at a concentration of 40.7 μM, and decreased AA-induced platelet aggregation significantly at middle concentration (81.3 μM) (Figure 6).

**Table 5.** Effects of dihydrocaffeic acid on New Zealand rabbit platelet plasma TT, APTT, PT (INR) (*n* = 6).

	Coagulation parameter		
Compound (concentration μM)	TT (S)	APTT (S)	PT (INR)
Control	10.00 ± 0.96	15.47 ± 0.83	1.00 ± 0.01
Dihydrocaffeic acid (85.9 μM)	11.33 ± 0.80	13.80 ± 1.32	1.03 ± 0.02
Dihydrocaffeic acid (171.7 μM)	9.63 ± 1.03	10.63 ± 0.58**	0.90 ± 0.05*
Dihydrocaffeic acid (343.4 μM)	9.37 ± 0.12	8.77 ± 0.35**	0.87 ± 0.03**

Data were expressed as mean ± SD, *n* = 6.

** *p <* 0.01 as compared to control group.

* *p <* 0.05 as compared to control group.

## Discussion

Normal physiological haemostasis and anticoagulation mechanisms are a complex physiological, biochemical and pathological process that consists mainly of interrelated three parts: vasoconstriction and platelet response, coagulation and anticoagulation systems and fibrinolysis systems (Furie and Furie 2008). In order to compare the different activities of ST and STC on blood circulation, some parameters were determined on the basis of normal and blood-bleeding rat models in the present study. Firstly, the bleeding rat model was established through deep subcutaneous injection of heparin sodium salt to rats. Different injection concentrations of heparin (100, 300 and 500 U/kg) were compared and characterized by rat bleeding time. It was found that the bleeding time of rats was significantly prolonged when given an injection of 500 U/kg of heparin, which was applied in further studies.

The tail-bleeding time was the most intuitive parameter to reveal the effectiveness for stopping bleeding. In this study, the effects of ST and STC on tail-bleeding time of rats were confirmed firstly. Although both ST and STC could not be effective as positive control YNBY, STC showed much stronger ability to shorten bleeding time than ST, which implied the haemostatic effect of STC to some extent. WBV was observed as one of the parameters to reflect the intrinsic resistance of blood to flow in vessels (Kesmarky et al. 2006). STC treatment could achieve significant increase of WBV at all shear rates, while ST treatment could not achieve it. At the meantime, PV as one important member in WBV was further detected. STC treatment was able to increase PV, which suggested that STC could increase WBV partly by increasing PV. Since red blood cells accounted for almost 50% of blood volume and constituted the majority of cellular content in blood, two common parameters include ESR and PCV used for characterizing the state of red blood cell in the whole blood were monitored. In this experiment, there was no significant difference between the administration group and model group for PCV. But in STC group, ESR significantly decreased, suggesting that the haemostatic effect of STC might be partly due to its influence on the number and volume of red blood cell. On the other hand, ST did not show any improvement of ESR and PCV as expected.

APTT, PT, TT and FIB are four commonly used blood indices to evaluate coagulation system, the four parameters could reflect the type and concentration of proteins in plasma. APTT and PT are the performance indicators for intrinsic and extrinsic coagulation pathways, respectively. Both FIB and TT reflected the function of fibrinogen in plasma. The present study showed that STC could significantly shorten APTT and showed no effect on PT and TT. ST shortened not only APTT, but also TT significantly, which suggested that ST might have anticoagulant characteristics. However, ST strongly decreased FIB content in plasma, while STC increased the FIB content to a relatively high level. These results illustrated that (1) both ST and STC exhibit some haemostatic potentiality through extrinsic coagulation pathway; (2) ST is possible to affect fibrinolysis system by antagonistic mechanisms; (3) FIB content might be the most important target for ST and STC, which could explain the final obtained bleeding time result. However, it was necessary to further investigate the specific mechanisms of ST and STC affecting coagulation parameters.

The potential compounds responsible for the different activity of ST and STC were investigated by HPLC analysis. It was found that the number and area of peaks in the HPLC chromatogram of STC has undergone obvious change as compared to ST. The accurate change of peak area was calculated and compared, finally total 25 peaks were considered to be the potential compounds (peaks) contributed to the different activities of ST and STC, and among them 11 compounds were tentatively identified.

In addition, dihydrocaffeic acid, one of the compounds with significantly increased peak area in STC, was selected as candidate for further haemostatic activity test. Interestingly, dihydrocaffeic acid (171.7 μM) significantly shortened APTT and PT, which partly fitted the activity of STC. Besides, dihydrocaffeic acid (< 350 μM) also influenced the platelet aggregation system, and significantly enhanced the collagen and trap-6 induced platelet aggregation. On the other hand, amentoflavone, reported for blood promoting function through vasodilative effect (Xu and Yin 2009), was the major component in ST. As a representative compound with significantly decreased peak area in STC, the anti-platelet aggregation effect of amentoflavone was also investigated. The significant inhibition effect of amentoflavone on ADP- and AA-induced platelet aggregation (< 100 μM) might suggest the blood promoting activity of ST. Although the further identification of structures and confirmation of activities for remaining different compounds would be considered to explore the transformation of activity of ST, the obtained activity result from dihydrocaffeic acid and amentoflavone could provide some supporting explanation for the specific activity of ST and STC.

## Conclusions

In this study, the different effects of ST and its carbonizing processed product STC on the physiological coagulation system were compared, and the potential active compounds responsible for their different activities were predicted according to the compositional variance. The results were showed that STC has strong haemostatic activity by shortening tail-bleeding time, increasing WBV and PV, decreasing ESR, reducing APTT and increasing FIB content, while ST has no influence on tail-bleeding time, WBV, PV, ESR and PCV. Through the HPLC-DAD and LC-MS analyses, total 11 distinguishing peaks were identified in ST and STC extracts. With increased amount of STC, dihydrocaffeic acid showed haemostatic activity for promoting the platelet aggregation and reducing APTT and PT. However, amentoflavone with decreased content in STC inhibited ADP- and AA-induced platelet aggregation. The data in this research implied that ST and STC function on the coagulation system with distinguishing effect and their compositional variance was attributed to this difference. These would be important references for clinical applications of ST and STC to bleeding or thrombotic diseases.

## References

[CIT0001] Chen C, Wang FQ, Xiao W, Xia ZN, Hu G, Wan JB, Yang FQ. 2017 Effect on platelet aggregation activity: extracts from 31 Traditional Chinese Medicines with the property of activating blood and resolving stasis. J Tradit Chin Med. 37:64–75. 29957905 10.1016/s0254-6272(17)30028-6

[CIT0002] Chen C, Yang FQ, Zhang Q, Wang FQ, Hu YJ, Xia ZN. 2015 Natural products for antithrombosis. Evid-Based Compl Alt. 2015:1–17. 10.1155/2015/876426 PMC4449941 26075003

[CIT0003] Chen GY, Han YD, He W. 2016 Amentoflavone protects against high fat-induced metabolic dysfunction: possible role of the regulation of adipogenic differentiation. Int J Mol Med. 38:1759–1767. 27748827 10.3892/ijmm.2016.2772 PMC5117752

[CIT0004] Committee, NP 2015 Pharmacopoeia of People’s Republic of China. Beijing: Chemical Industry Press.

[CIT0005] Dahlback B. 2000 Blood coagulation. Lancet. 355:1627–1632. 10821379 10.1016/S0140-6736(00)02225-X

[CIT0006] Davie EW, Fujikawa K, Kisiel W. 1991 The coagulation cascade: initiation, maintenance, and regulation. Biochemistry-US. 30:10363–10370. 10.1021/bi00107a001 1931959

[CIT0007] Farombi EO, Owoeye O. 2011 Antioxidative and chemopreventive properties of *Vernonia* amygdalina and *Garcinia* biflavonoid. Int J Environ Res Public Health. 8:2533–2555. 21776245 10.3390/ijerph8062533 PMC3138040

[CIT0008] Furie B, Furie BC. 2008 Mechanisms of disease: mechanisms of thrombus formation. N Engl J Med. 359:938–949. 18753650 10.1056/NEJMra0801082

[CIT0009] Jung YJ, Lee EH, Lee CG, Rhee KJ, Jung WS, Choi Y, Pan CH, Kang K. 2017 AKR1B10-inhibitory *Selaginella tamariscina* extract and amentoflavone decrease the growth of A549 human lung cancer cells *in vitro* and *in vivo*. J Ethnopharmacol. 202:78–84. 28286104 10.1016/j.jep.2017.03.010

[CIT0010] Kesmarky G, Feher G, Koltai K, Horvath B, Toth K. 2006 Viscosity, hemostasis and inflammation in atherosclerotic heart diseases. Clin Hemorheol Microcirc. 35:67–73. 16899908

[CIT0011] Lee CW, Kim HS, Choi HJ, Kim JW, Kim HK, Moon HT, Woo ER. 2008 Biflavonoids isolated from *Selaginella tamariscina* regulate the expression of matrix metalloproteinase in human skin fibroblasts. Planta Med. 74:1006–1006. 10.1016/j.bmc.2007.10.036 18029185

[CIT0012] Lee J, Choi Y, Woo ER, Lee DG. 2009 Antibacterial and synergistic activity of isocryptomerin isolated from *Selaginella tamariscina*. J Microbiol Biotechnol. 19:204–207. 19307771 10.4014/jmb.0810.566

[CIT0013] Li GL, Wei SH, Zhang ZL, Li HB. 2011 Comparison of contents of total flavonoids in different processed products of *Selaginella*. China J Chinese Med. 26:194–195.

[CIT0014] Mackman N. 2008 Triggers, targets and treatments for thrombosis. Nature. 451:914–918. 18288180 10.1038/nature06797 PMC2848509

[CIT0015] Nguyen PH, Ji DJ, Han YR, Choi JS, Rhyu DY, Min BS, Woo MH. 2015a Selaginellin and biflavonoids as protein tyrosine phosphatase 1B inhibitors from *Selaginella tamariscina* and their glucose uptake stimulatory effects. Bioorgan Med Chem. 23:3730–3737. 10.1016/j.bmc.2015.04.007 25907369

[CIT0016] Nguyen PH, Zhao BT, Ali MY, Choi JS, Rhyu DY, Min BS, Woo MH. 2015b Insulin-mimetic selaginellins from *Selaginella tamariscina* with protein tyrosine phosphatase 1B (PTP1B) inhibitory activity. J Nat Prod. 78:34–42. 25559759 10.1021/np5005856

[CIT0017] Peng ZC, Zhang SW, Liu Y, Liang YH. 2000 The influence of *Selaginella tamariscina* on hemostasis after stir-frying process. China J Chinese Materia Med. 25:89–90.

[CIT0018] Stenberg PE, Barrie RJ, Pestina TI, Steward SA, Arnold JT, Murti AK, Hutson NK, Jackson CW. 1998 Prolonged bleeding time with defective platelet filopodia formation in the Wistar Furth rat. Blood. 91:1599–1608. 9473225

[CIT0019] Suh SJ, Chung TW, Son MJ, Kim SH, Moon TC, Son KH, Kim HP, Chang HW, Kim CH. 2010 Erratum to The naturally occurring biXavonoid, ochnaXavone, inhibits LPS-induced iNOS expression, which is mediated by ERK1/2 via NF-kappa B regulation, in RAW264.7 cells (vol 447, pg 136, 2006). Arch Biochem Biophys. 493:249. 10.1016/j.abb.2006.01.016 16527246

[CIT0020] Tang L, Chen YC, Jiang ZB, Zhong SP, Chen WZ, Zheng FC, Shi GG. 2017 Purification, partial characterization and bioactivity of sulfated polysaccharides from *Grateloupia livida*. Int J Biol Macromol. 94:642–652. 27773841 10.1016/j.ijbiomac.2016.10.067

[CIT0021] Wang JM, Lian PL, Yu Q, Wei JF, Kang WY. 2017 Antithrombotic mechanism of polysaccharides in blackberry (*Rubus* spp.) seeds. Food Nutr Res. 61:1379862 29056892 10.1080/16546628.2017.1379862 PMC5642186

[CIT0022] Weng JK, Noel JP. 2013 Chemodiversity in *Selaginella*: a reference system for parallel and convergent metabolic evolution in terrestrial plants. Front Plant Sci. 4:119. Last page? 23717312 10.3389/fpls.2013.00119 PMC3650682

[CIT0023] Xie PY, Zhang Y, Wang XB, Wei JF, Kang WY. 2017 Antithrombotic effect and mechanism of *Rubus* spp. blackberry. Food Funct. 8:2000–2012. 28485425 10.1039/c6fo01717g

[CIT0024] Xu DY, Huang P, Yte ZS, Xing DH, Ouyang S, Xing GQ. 2015 Efficacy and safety of *Panax notoginseng* saponin therapy for acute intracerebral hemorrhage, meta-analysis, and mini review of potential mechanisms of action. Front Neurol. 5:1–19. 10.3389/fneur.2014.00274 PMC4288044 25620952

[CIT0025] Xu L, Yin MH. 2009 Experiment study on vasodilative effects of amentoflavone ethyl acetate extract of *Selaginella tamariscina*. Yanbian Univ Colledge Trad Chin Med. 32:246–248.

[CIT0026] Yang RF, Wu SH, Wei SH, Zhang ZL, Li HB, Li GL. 2015 The effects of *Selaginella tamariscina* and *Selaginella tamariscina* Carbonisatus on blood bleeding time and coagulation time in mice. Tradit Chin Med Res. 28:70–73.

[CIT0027] Yang SZ, Flaws B. 1998 The divine farmer's material medica: a translation of the Shen Nong Ben Cao Jing. 1st ed. Boulder CO: Blue Poppy Press.

[CIT0028] Zhang YX, Li QY, Yan LL, Shi Y. 2011 Structural characterization and identification of biflavones in *Selaginella tamariscina* by liquid chromatography-diode-array detection/electrospray ionization tandem mass spectrometry. Rapid Commun Mass Spectrom. 25:2173–2186. 21710597 10.1002/rcm.5090

